# The Role of Temporality in Virtual Reality Interventions for Depressive Episodes—A Scoping Review

**DOI:** 10.3390/healthcare14020156

**Published:** 2026-01-07

**Authors:** Volha Saroka, Tomir Jędrejek, Marcin Trybulec, Zuzanna Aleksandra Rucińska

**Affiliations:** 1 Faculty of Philosophy and Sociology, Maria Curie-Skłodowska University, 20-031 Lublin, Poland; jedrejektomir@gmail.com; 2 Centre for Philosophical Psychology, University of Antwerp, 2000 Antwerpen, Belgium; zuzannaaleksandra.rucinska@uantwerpen.be

**Keywords:** temporality, virtual reality, depression, mental health

## Abstract

**Highlights:**

**What are the main findings?**

- The temporal dimension in VR-based interventions for depression is important, yet underrepresented in the literature.
- Two features are considered essential in supporting the reorganization of the experience of time in depression through VR: generating immersion and scaffolding vivid imagination through visualization.
- VR has the capacity to generate experiences otherwise inaccessible in real life, such as shifting between perspectives and enabling interaction with abstract constructs, which remains underrated.

**What are the implications of the main findings?**

- VR-based interventions may benefit from engaging individuals more actively through designing interactive experiences that provide opportunities for action.
- Drawing on VR’s underrated features such as interaction with abstract entities and possibility to switch between first-, second- and third person perspective consist a promising directions for further research.
- Since all analyzed studies are exploratory, further research using more coherent experimental designs is needed.

**Abstract:**

**Background/Objectives:** People living with depression often experience consistent disruptions in their experience of time, which further contributes to their suffering. We present a scoping review on virtual reality (VR)-based interventions for depression, addressing temporal processing and subjective experiences of time. The paper aims to explore the extent to which therapeutic interventions using VR target the temporal dimension of patients’ experiences. **Methods:** We conducted a scoping review using the PRISMA 2020 standard. The literature search was further extended using Research Rabbit and by examining the reference lists of relevant articles. Seventeen papers were selected for final analysis. **Results:** Our scoping review indicates that temporality in VR-based therapeutic interventions for depression remains underrepresented. Of the seventeen papers reviewed, only two explicitly deal with this issue, while the rest touch upon it briefly or implicitly. The studies suggest that VR’s main advantage in modifying the experience of time in depression is its potential to generate immersion and to scaffold imagination through visualization. The main limitations are methodological: most of the available research is exploratory, reports short-term effects, and utilizes a broad variety of empirical designs and therapeutic approaches.

## 1. Introduction

The World Health Organization states that globally, an estimated 5.7% of adults suffer from depression [1]. Even less than half receive mental health support and treatment. This highlights the need for innovative, cost-effective, and accessible interventions.

According to the DSM-5-TR, depression involves symptoms spanning three core domains: emotional (e.g., persistently low mood), cognitive (e.g., difficulty in concentrating or making decisions), and somatic or psychosomatic (e.g., insomnia, changes in appetite, or chronic fatigue). Although the DSM-5-TR does not explicitly mention temporal disruptions as diagnostic criteria, alterations in the subjective experience of time are significant and well-documented aspects of the depressive disorder within phenomenological psychiatry [2,3,4,5]. Depressed individuals report a slowing of time, an overwhelming sense of the past, a frozen experience of the present, and a future that feels closed off. Distortions in time experience should be treated not only as symptoms of depression, but also as therapeutic targets that virtual reality (VR) is particularly well-suited to address.

The body of research has pointed to VR as one of the promising technologies in mental health care that can supplement traditional face-to-face interventions [6,7,8]. Unlike traditional media, VR enables immersive engagement, eliciting a sense of presence and agency within a simulated environment. In discussions on media-based treatments, VR is often praised for its potential to create immersive environments, alongside smartphone applications, extended-reality and online interventions [9,10,11], making it a valuable therapeutic tool. It allows the therapists to create realistic and safe scenarios, where patients can confront distressing experiences, relive positive or negative memories, and practice a variety of coping strategies. Additionally, VR makes it possible to simulate experiences that are not attainable in real-world settings. According to Bell et al. [8,12] VR enhances the therapeutic process because it can be personalized and tailored to the needs of each individual. VR can also serve as an intermediate step between social withdrawal and reintegration into everyday life. Importantly, VR provides a fully controllable environment. In case of discomfort, the session can be paused at any moment, and the therapist can regulate the intensity of stimuli to align with the client’s therapeutic progress. Additionally, standard methods may sometimes be less effective because they do not evoke the same level of experiential impact that immersive, interactive VR environments can generate.

In addition to the aforementioned benefits in the field of mental health in general, there are indications that VR could be particularly well suited for therapeutic interventions in depression. First, VR can enhance engagement through gamification [13], which is especially valuable in depression, where motivation and interest are often diminished. Gamification can involve physical exercises, social activities, or behavioural skills development. VR can increase the level of engagement because virtual experiences are perceived as extraordinary and less demanding. Moreover, patients perceive activities performed in VR as less exposed to critical evaluation by others [14,15]. Second, by encouraging active engagement, VR game mechanics can strengthen patients’ sense of agency, a capacity that is frequently reduced in depression [16]. One’s sense of agency can be increased because 3D VR environments afford a wider range of activities than standard PC-based gaming interventions. While people in depression struggle to initiate activity and to envision meaningful future actions, VR environments allow even minimal movements (e.g., gentle hand gestures) to produce visible, satisfying and often amplified changes in the virtual world [16]. Third, VR environments produce affective outcomes that can be tailored to a person’s needs [17]. For example, a peaceful virtual forest by encouraging participants to focus on the present moment rather than on distressing past experiences, enables mindfulness practices.

Although many studies point to the effectiveness of VR in supporting mental health [18,19,20,21], the review by Freher et al. [16] points to the fact that the topic of VR interventions in depression, is still in its early stages. A systematic review provided by Wiebe et al. [22] shows that only 18 studies were concerned with the use of VR in depression, compared to 91 studies on schizophrenia spectrum disorders and 159 on anxiety disorders. Existing reviews tend to focus on broad mental health domains rather than depression specifically. For example, Bell et al. [12] provide an overview focusing on the mechanisms of effective VR interventions, which includes a broad range of mental disorders aside depression, such as anxiety, psychotic symptoms, PTSD, eating disorders, or stress management.

In addition to the fact that VR in depression has been understudied, temporality has appeared even less frequently, and when it has been noted, it has not been the main target of therapeutic intervention. VR would be particularly well-suited to address temporal distortions, because VR environments allow alterations of the experience of time and space. For instance, one can create temporal experiences that may be impossible to achieve in the physical world, and adapt them to individual needs. Thus, while an increasing number of studies explore the use of VR in the treatment of mental disorders [12,16], including depression, little attention has been devoted to its potential to modulate the perception of time. In fact, to date, no systematic review has examined VR-based interventions that specifically address the temporal dimension of depression.

Our scoping review seeks to address this gap by organizing and critically analyzing existing research on time-related dimensions of VR interventions for depressive disorders. We describe the scope of the gaps in the literature, and organize existing studies according to the time dimensions to which they refer. We then present the mechanisms considered to be essential to supporting the reorganization of time perception in depression through VR. Finally, we highlight the limitations of existing studies, and suggest directions for future research.

Before we begin, we will address a caveat on the terminology used in this article. In the reviewed studies, VR is defined as a computer-generated, three-dimensional environment that provides multisensory input and enables users to interact directly with virtual objects [23]. Immersion is defined as a psychological state of deep engagement emerging from the interaction between the user and the 3D environment. It encompasses a cognitive and emotional absorption in the virtual environment [24]. Flow is defined as a state of deep engagement elicited by activities characterized by clear, attainable goals, a balance between challenges and skills, and rewards [25]. Notably, both immersion and the experience of flow can distort time perception; however, they do so in distinct ways. While the optimal experience during a state of flow leads to a subjective shortening of how time is experienced (“speeding up” of time), immersion can have the opposite effect, enhancing a focus on the present moment and either a subjective “deceleration” or “accelaration” of time. The subsequent analyses will distinguish between studies focusing primarily on immersion and those examining the flow state induced by VR.

## 2. Experience of Time, Depression and VR Interventions—An Overview

### 2.1. Experience of Time in Depression

Ratcliffe aptly describes first-person experience of time in depression as follows: “When depressed, time seems to slow down, and at a certain point can become irrelevant. It is easy to lose track of days without realising it” [26] (p. 175). The experience of time in depression is characterized by a deceleration of temporal flow [27,28,29]. Changes in the subjective experience of time are corroborated by empirical findings, which show that patients with depression tend to overestimate time intervals in objective temporal judgement tasks [30,31,32]. The past also takes on particular significance. Depressed individuals often experience the dominance of the past, with its losses and failures, over the future and its possibilities [4]. As Gallagher [28] indicates, individuals with depression are frequently preoccupied with past experiences while showing diminished engagement with the present and the future. The past is characterized as unchangeably negative [2], overwhelming and discontinuous, where perception of linear time is blocked [33]. The present moment is experienced as meaningless and reduced to repetitive circling, and the time in the present feels suspended [2]. For instance, Fusar-Poli et al. found that the patients typically declare that they “can’t remember days because time has stopped” [5] (p. 357). Moreover, the present is experienced as stagnation, whereas the future appears to be “impossible” or predetermined [26,34]. It carries hopelessness, and no possibility of change. To speak in Minkowski’s words, “the future orientation, which gives meaning and direction in life, is missing” [35] (p. 301). In short, in depression, individuals are reported to experience loss of goal-directedness and drive, which leads to a slowing down, and, eventually, a “freezing” of lived time.

Fuchs [4] also proposed a strategy to show how to adapt lived temporality in depressive patients to the rhythms of the external environments. It involves five stages of working with the patient. First, creating a peaceful space to help in recovery; second, introducing basic regularity into daily life; third, gently engaging the patient to restore their orientation toward the future; fourth, gradually increasing the level of stimulation, tailored to the personal needs; and finally, rebuilding social relationships. In Section 6 (Discussion and future directions), we will return to Fuchs’ analytical framework to assess which stages of his resynchronisation therapy are represented in our collected studies (below), and how VR technologies may supplement them.

### 2.2. VR Therapy for Depression

A systematic review of VR interventions in emotional disorders [36] highlights that Cognitive Behavioural Therapy (CBT) and its sub-type exposure therapy are the leading methods of VR therapy for depression. The frequency of appointments and the duration of VR use depend on the needs of the person, typically starting with a 20–90 min session [37]. On average, patients receive eight weekly sessions [38]. Prior to the session, the therapist provides the patient with an explanation of the VR intervention, and following the session, they discuss the patient’s experience.

Lindner et al. [39] showcase how psychoeducation, behavioural activation, social skills training, cognitive restructuring, avatar therapy and virtual gardening can be integrated with CBT-VR. Freher et al. [16] indicate that VR therapy can enhance positive affect, mental imagery, and improve cognitive functioning of depressive patients. VR can also be used for neurofeedback [40], group therapy [14], or simulating the phenomenological aspects of psychedelic and mystical experiences [41]. VR technologies can further address depressive symptoms through gaming interventions, whose narrative and point-collection aspect fosters sensorimotor activation and positive emotions [42]. However, there is still much less research on using VR for depression, compared to research on the use of VR for post-traumatic stress disorder (PTSD) and anxiety disorders [43,44]. The majority of research on the role of VR for depression is individually designed, which suggests that this field remains relatively underdeveloped [16,36].

### 2.3. VR Interventions and Time Experience

VR technology is a promising tool to investigate factors influencing subjective time perception. The advantage of VR lies in its capacity to create simulated spaces and regulate sensory output. Under typical conditions, using VR leads to faster subjective passage of time in comparison with non-VR conditions [45,46]. Time experience can be manipulated in VR through visual and auditory means. For instance, Rogers et al. [47] showed that music in VR correlates with underestimation of the time spent in VR. The degree of avatar representation in VR (so-called “VR embodiment”) also played a role in subjective time experience [48,49]. Lower levels of embodiment (no avatar) resulted in the experience of slowing down the passage of time, in comparison to partial and full-body representations [50,51,52]. The negative effects associated with experiencing VR, such as simulator sickness, further dilated the subjective experience of time, and lead to an overestimation of temporal intervals [53].

A body of research explored how the motion of virtual objects and the illusion of self-motion influences temporal perceptions [53,54,55]. The studies employed the so-called “virtual zeitgebers” or chronometers, which are external cues that aid in the tracking of time. Examples of zeitgebers include a pendulum or the movement of the Sun [54]. For instance, Schatzschneider et al. [54] found that a static virtual sun caused distortion of time estimation, whereas natural or accelerated motion improved temporal accuracy. Ozyurt and Ikhwan [55] demonstrated that scaling up grid patterns in virtual landscapes significantly decreased the subjective passage of time. Smaller spatial scales intensified the sense of self-motion and produced a faster perception of time flow. Taken together, these findings demonstrated that VR provides a useful tool for manipulating subjective time perception, and has the potential to inspire therapeutic approaches for depression.

## 3. Materials and Methods

This study applied the Preferred Reporting Items for Systematic Reviews and Meta-Analyses (PRISMA) 2020 methodology to explore research on VR-based interventions for depression that addressed subjective experience of time. This study has been registered on the Open Science Framework (Registration DOI: 10.17605/OSF.IO/2F6G4) on 25 November 2025.

We proceeded in three steps. First, we defined three inclusion criteria for the study selection. During each stage of the search and screening process, texts were selected for further analysis if they satisfied one or more of the following criteria:

(1) address the modulation of the experience of time through VR interventions and directly or indirectly refer to episodes associated with depressive disorders;
(2) address the use of VR interventions for individuals with depressive disorders and directly or indirectly refer to aspects of the experience of time;
(3) address the temporal experience in the context of depressive disorders and directly or indirectly refer to the application of VR technologies.

Second, we conducted a systematic search of the Scopus and PubMed databases following the PRISMA guidelines. In order to ensure the alignment with field-specific terminology, we used the following keywords: “Virtual Reality” (VR), “extended reality”, “VR intervention”, and “VR therapy”, as well as “Depression*” (depressive disorder; melancholia), and “Time” (time experience*; time perception*; lived time; subjective time; temporality; temporal experience; time disturbances). We expanded the search by combining these core terms with supplementary descriptors such as “treatment”, “first-person”, “qualitative*”, and “phenomenology*”. There were 189 combinations of keywords. The review included publications available in English up to August 2025. 

During the initial search, a total of 3546 records were identified, including 2087 from Scopus and 1459 from PubMed. After removing duplicates and irrelevant records, 846 articles remained. Subsequently, the records were screened by title and abstract according to the predefined inclusion criteria. At this stage, 812 articles were excluded as they did not sufficiently correspond to the research focus, resulting in 34 articles retained for full-text review. The selection of sources followed a multi-stage screening according to predefined eligibility criteria. For each article, reviewers extracted key information including type of VR intervention, target population, reported effects, depression-related outcomes, and the degree to which time-related processes were addressed. Three reviewers independently assessed whether a study met the inclusion criteria. Studies receiving at least two inclusion votes advanced to the final stage of analysis. Upon closer examination, 22 out of 34 studies were excluded. Consequently, 12 articles were identified from Scopus and PubMed databases as relevant and included in the review. To ensure reproducibility in the study selection and data extraction, we operationalized time-related processes using three categories: (1) time perception (judgments of duration, passage-of-time evaluations, and perceptual time distortions); (2) lived temporality and temporal structure (altered temporal orientation, experiences of being “stuck-in-time”, disruptions in temporal structure); (3) temporal orientation (references to past, present, and future; anticipation, planning, rumination).

**Figure 1:**
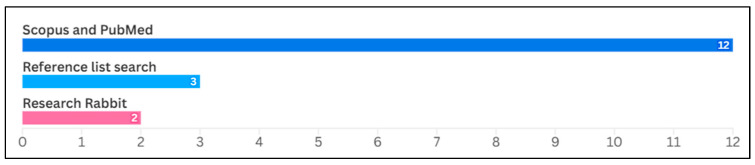
Selected studies by literature source.

**Figure 2:**
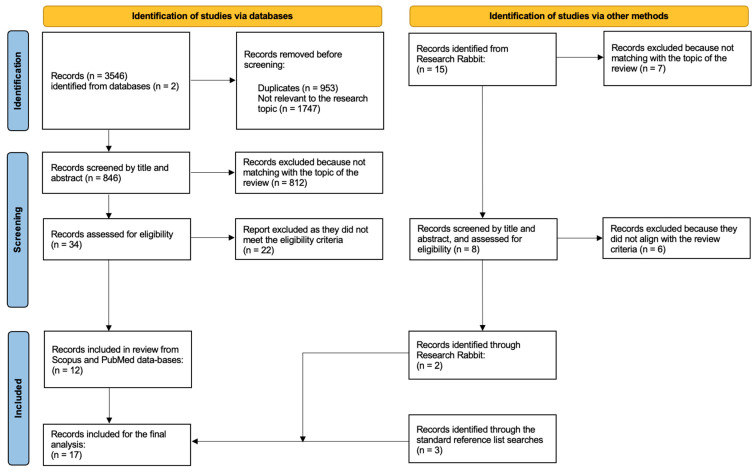
Selection process for the scoping review.

Third, the PRISMA search resulted in a relatively small number of largely overlapping studies. Therefore, we decided to supplement it using the Research Rabbit literature-mapping tool and with a standard review of the reference lists of relevant papers. Unlike PRISMA, Research Rabbit allows for key-word free search, permitting us to consider a broader research context. We used the twelve previously selected papers (through the PRISMA procedure) as a basis for the Research Rabbit exploration of connected papers. This resulted in the selection of an additional 15 records. Out of the 15, we chose two articles that supplemented the PRISMA-based dataset. Additionally, a bibliography review of the collected papers identified three additional studies. Finally, all researchers met to reconcile decisions and confirm the final set of studies included in the scoping review. The bar chart (Figure 1) below summarizes the distribution of the selected papers across the different data sources. Ultimately, 17 papers were included in the final analysis (see Figure 2).

There are two main reasons why a relatively small number of papers was identified through the PRISMA search. The first is that the topic of temporality in VR therapy for depression is a niche area. The second is that the studies relevant to our interest often employ diverse terminology that is not fully captured by initial search terms. This justifies the use of Research Rabbit, which visualizes connections between publications, regardless of keywords. It reduces the risk of selection bias, given that the search conducted in PubMed and Scopus resulted in a strongly clustered set of papers. 5 out of 12 studies selected in PRISMA search originate from authors associated with the VIRTUALTIMES project [49,50,56,57,58].

## 4. The Research Landscape

Data was charted using a set of extraction criteria collaboratively developed by three researchers. Each researcher independently generated an initial list of variables relevant to analyzing VR interventions for depression and their relationship to time-related processes. Their findings were then compared and consolidated into a single calibrated extraction form, containing only the criteria shared across all three lists. The final extraction form was used for all included studies and contained the following fields: (1) Author(s) and year of publication; (2) Clinical condition targeted; (3) Therapeutic strategy applied; (4) Description of the VR intervention; (5) Temporal dimension addressed by the intervention (past, present, or future); and (6) Reported psychological effects related to time. The form was reviewed by the team before data charting began to ensure clarity and completeness. Table 1 below organizes the collected studies according to the enlisted criteria.

In order to organize the collected material, we used a grounded theory to develop bottom-up analytical categories [59] (pp. 315–318+405–406). Through systematic reading and an analysis conducted by two independent coders, we identified three basic overarching thematic clusters focusing on the aspects of time experience in VR-based interventions for depression. The first cluster comprises four studies of VR-based interventions that support patients’ engagement with the present (Section 4.1). The second cluster included four studies concerned with VR-based interventions that also engaged patients in the present, but helped them in redefining their attitude toward the future (Section 4.2). The largest cluster of studies comprised nine papers focused on ways of manipulating the experience of time through VR technology. These studies explored how virtual environments can accelerate or decelerate time perception, justifying their importance by reference to possible therapeutic application for depressive disorders (Section 4.3).

Table 1 shows that eight papers were concerned with various forms of depressive disorder, while the remainder focused on manipulating time through VR. This cluster of research mentioned the therapeutic use of VR as the rationale for the research. Consequently, no therapeutic methods were assigned to this cluster of research in Table 1. The remainder of the studies varied significantly in the way the therapeutic interventions were designed. We use the classification of therapeutic intervention methods proposed by Freher et al. [16] to show that none of them were dominant.

Moreover, the data in Table 1 demonstrate that all interventions are related to the present. The studies supported the patients through generating meaningful and pleasant moments, and modifying the pace of their experience of time. Five studies used positive experiences in the present to alter patients’ attitudes towards the past or future. These effects were achieved thanks to the three main features of VR: immersion, sense of presence, and physical interaction capabilities. While all VR interventions were constructed to create a sense of immersion and presence, designs based on VR’s interaction possibilities are rather scarce. Immersion and sense of presence are used to evoke positive emotions that aim to strengthen patients’ motivation to take action and encourage regular participation in therapy. Only six studies have employed the VR’s capacity to facilitate direct interaction with virtual objects (e.g., by whole-body or hand movements). Three interventions used interactive features of VR to a minimal extent (e.g., guided meditation, or exploring a 3D landscape with VR controllers). The remaining studies applied exposure or waiting scenarios and did not take advantage of VR’s interactive capabilities.

**Table 1:** Studies characteristics and psychological effects regarding time in papers, included for analysis.

No.	Author	Clinical Condition	Therapeutic Strategy	Description of VR Intervention	Time Dimension	Psychological Effects Regarding Time
1	Buele et al. (2024) [60]	MDD	Skills training Enhancing cognitive functioning	Real life motor training supported by VR-based cognitive training involving simulated daily life tasks.	Present	Improved executive functions, spatial and temporal orientation.
2	Colombo et al. (2022) [61]	MDD	Behavioural activation Psychoeducation	Behavioural activation (BA) protocol using VR. Subjects participated in favourite activities through Google Earth VR.	Present Future	Moderate to large increase in the time spent planning and engaging in the activities scheduled during the intervention.
3	Fernandez-Alvarez et al. (2021) [62]	MDD	Mental imagery	Participants recalled positive autobiographical memory using Google Earth VR app.	Present Past	VR used to provide a spatial reference and evocative environment for recalling positive memories, reducing rumination.
4	García-Gutiérrez et al. (2025) [63]	MDD	Cognitive restructuring	Participant used EYME digital platform in VR to visualize and imagine personal cognitive schemas and shape self-perception.	Present Future	Enhancing positive future oriented imagining.
5	Huang et al. (2022) [64]	MDD	Enhancing cognitive functioning	Working Memory Training (WMT) through VR app (e.g., supermarket shopping, flowerpot replenish).	Present Future	VR-based working memory training indirectly boost event-based prospective memory performance
6	Igarzábal et al. (2021) [56]	None	None (Time perception manipulation)	Participants waited for 7.5 min in a VR room.	Present	Participants in VR were more bored and experienced a slower passage of time compared to the real waiting room scenario.
7	Ke et al. (2024) [65]	None	None (Time perception manipulation)	Participants performed a cycling task while exposed to surrounding exercising avatars in a VR gym.	Present	Faster self-motion in VR and higher exercise intensity of avatars accelerated subjective passage of time and, to a certain extent, time duration judgments.
8	Landeck et al. (2023b) [57]	None	None (Time perception manipulation)	Participants were exposed to objects displaying different motion types (e.g., pendulum).	Present	Effects of the motion of virtual zeitgebers on time duration estimation and subjective passage of time.
9	Landeck et al. (2023a) [49]	None	None (Time perception manipulation)	Participants were exposed to the motion of the virtual tunnel.	Present	Effect of the density and speed of a virtual tunnel on the illusion of self-motion and the perception of time.
10	Landeck et al. (2024) [58]	None	None (Time perception manipulation)	Participants were exposed to virtual zeigebers (clock and orbit pendulum).	Present	Effect of different types of motion (slow/medium/fast and regular/irregular) of virtual zeitgebers on time duration estimation and subjective passage of time.
11	Mao et al. (2024) [66]	MDD	Skills Training	Mindfulness Training in VR app that supported a personalized course, intelligent monitoring, emotion tracking, and games.	Present	Multisensory VR scenarios with meditative music enhanced immersion, helping patients focus on the present moment and expand body awareness.
12	Miller et al. (2023) [67]	MDD	Psychoeducation Behavioural activation Skills training	Self-guided VR-CBT application supporting behavioural activation, mood tracking, mindfulness and activity scheduling, problem-solving and continued activity	Present Future	Mood tracking and activity scheduling via VR app increased sense of control, emotional regulation and time structuring. Activity scheduling helped to impose structure on time.
13	Olasz et al. (2024) [68]	MDD	Skills Training	Mindfulness session on a VR beach with voice-guided breathing, body scan, and visualization to engage imagination.	Present	VR-mediated mindfulness exercises accelerated the perceived passage of time. The effect of acceleration is linked to immersion and flow state.
14	Rutrecht et al. (2021) [69]	None	None (Time perception manipulation)	Participants played a fast-paced game *Thumper* in both VR and desktop versions. Difficulty progressively increased.	Present	The flow state generated faster subjective perception of time, but did not significantly influence the underestimation of time duration.
15	Unruh et al. (2024) [70]	None	None (Time perception manipulation)	VR used to elicit an Out-Of-Body experience. Participants experienced a transition from the first- to third-person perspective in VR.	Present	Disembodiment and transition from first- to a third-person perspective affect time duration estimations and subjective experience of time passage.
16	Unruh et al. (2021) [50]	None	None (Time perception manipulation)	Waiting in a real room vs. a VR room (avatar and no-avatar conditions).	Present	Effect of virtual embodiment on time duration estimation and subjective passage of time.
17	Wang et al. (2025) [71]	None	None (Time perception manipulation)	1 min VR scenes featuring either evocative or conventional content.	Present	Evocative VR environments lead to faster experienced passage of time.

Our analysis indicates that the use of VR can sometimes produce unexpected outcomes. For instance, waiting in VR may induce greater boredom and lead to a slower subjective passage of time than anticipated [50,56]. These findings suggest that VR is not a universal solution that automatically produces desired results. Instead, it can lead to unexpected outcomes depending on factors such as the level of embodiment, the type of task, or participant expectations and cultural practices. We will now present our findings on the three thematic clusters focusing on the aspects of time experience in VR-based interventions for depression.

### 4.1. Engaging the Present

The four studies below show the capacity of VR to support patients in focusing on the present, maintaining positive experiences, and enhancing imaginative vividness of memories. In particular, VR mindfulness and cognitive training increased positive mood and motivation with reduction in anxiety and rumination levels.

Mao et al. [66] explored how VR-based mindfulness training helps patients with depression, anxiety, and patients undergoing chemotherapy with cancer-related fatigue. 48 participants completed four weeks of VR-based mindfulness training. The personalized course comprised seven submodules, including mindfulness breathing, awareness of thoughts, perception of sound, body scanning, immersive stress reduction, and deep relaxation. The results indicate that one week intervention reduced anxiety and depressive symptoms. This study is relevant for lived experience of time, as VR-based mindfulness encourages patients to focus on the present moment.

Olasz et al. [68] compared the effectiveness of a mindfulness exercise, delivered via VR and a tablet device. The Guided Meditation VR™ application delivered a 20 min relaxation programme set in a virtual beach environment without background music. According to Olasz et al. [68], both VR and tablet interventions were equally effective in reducing anxiety. However, participants in the VR condition perceived the sessions as shorter than their actual duration, compared to the tablet condition. This effect may be due to the state of immersion and flow induced by the VR, as indicated by other studies in our review [56,69]. Experiencing the flow state also facilitates the adherence to therapeutic practice, offering indirect benefits for dealing with chronic disorders, such as depression. Mindfulness training has been shown to increase awareness of the present moment, thereby reducing distress.

Buele et al. [60] designed a study to assess efficacy of dual intervention (motor training and VR-based cognitive exercises) for 34 elders (aged 65 and above), with mild cognitive impairment and depressive symptoms. The research utilized a pre-test/post-test randomized controlled trial design. An experimental group was engaged in VR-based cognitive training, while a control group executed conventional cognitive training. Training in VR simulated routine tasks, such as searching for ingredients in a virtual kitchen. It was designed to target memory, attention, executive functions, and time-space orientation. After six weeks and twelve sessions with motor and cognitive training, there was an improvement in cognitive abilities, and depressive symptoms were reduced in both the experimental and in the control groups. No advantage of VR over traditional cognitive training was observed. The experimental group exhibited higher motivation and a completion rate compared to the control group. Temporal orientation has been mentioned as one of the indicators of enhanced cognitive abilities, but it has not been examined as an independent factor.

Fernandez-Alvarez et al.’s [62] study showed how VR can support autobiographical memory and emotion regulation in patients with major depressive disorder (MDD). The study used single-case multiple baseline design. Eighteen adults, during two sessions in Google Earth VR, visited previously identified places associated with their pleasant experiences (e.g., a beach or a park). While in VR, the participants were asked to verbalize their memories associated with the scenery. Immersion in the virtual environment not only enhanced the recall of positive memories, it made them more imaginatively vivid and detailed in the moment of the VR experience. Participants reported an increased positive mood, as well as reduced rumination. However, this effect gradually faded after about a week. Reviving positive memories in the present was intended to help patients re-establish new relation to their past. The authors of the study indicated that a similar procedure could be applied when working with the future, for instance, by creating a space that allows individuals to engage in future-oriented imagining or planning. This approach was employed in the studies described in the next section as well.

### 4.2. From Engaging the Present to Orienting Toward the Future

The four studies below focus on the role of VR interventions in allowing the patients diagnosed with depression to focus on the present moment and future-oriented imagining: planning, prospecting and imagining future self.

Colombo et al. [61] performed an exploratory study with VR supported behavioural activation (BA). The aim was to enhance engagement in meaningful daily activities among seven participants with a depressive disorder. The study applied a single-case experimental design with multiple baselines. During a two-week intervention, patients benefited from four sessions with Google Earth VR, where they visited personally significant places, associated with pleasant experiences (e.g., a park). They imagined, visualized, planned, and rehearsed future activities in an immersive VR environment, guided by designed therapeutic narration. Results showed gradual improvements in daily activity, planning, savouring, and mood. The intervention influenced participants’ lived temporalities by fostering positive attitudes toward the present, and imagining a hopeful future. The authors reported that the sense of presence and immersion offered in VR supported the patients’ motivation and emotional engagement, and enabled vivid imaginings of future joyful activities.

Miller et al. [67] evaluated the feasibility and efficacy of a self-guided CBT-based digital intervention, by combining a mobile app with VR experiences for adolescents with depressive symptoms. 30 participants completed five modules over five weeks at their own pace. Each module included app components accompanied by a VR experience, such as immersive educational videos on depression and behavioural activation, mindful breathing, and guided meditations on problem-solving and relapse prevention. Participants reported that the programme helped them to structure time for their activities, make choices, plan, and imagine enjoyable future actions. Both quantitative and qualitative data supported the intervention’s acceptability and efficacy for delivering self-guided therapeutic content; significant reductions in depressive symptoms were observed.

Huang et al. [64] explored how VR-based working memory training (WMT) could improve event-based prospective memory. Their study involved the present and future dimensions of time. 46 participants with MDD were divided into experimental and control groups, and compared with 41 healthy individuals. Participants in the VR group completed 20 sessions of VR-based WMT. The VR-based programme consisted of five memory tasks (e.g., memorizing a shopping list, or remembering the positions of flowerpots). The findings indicated that VR-based WMT enhanced event-based memory of intentions directed towards the future.

García-Gutiérrez et al. [63] presented a case study involving ten therapeutic sessions (one hour a week), using a VR platform Explore Your Meanings (EYME), to support identity reconstruction for a 21-year-old woman with MDD and social phobia. The EYME enabled users to visualize, imagine and interact with different dimensions of self-identity. The intervention implicitly involves lived temporality by addressing the tension between the patient’s present and ideal future selves, and enhancing her optimism toward the future. Users could define the present self (“Who am I?”), the ideal self (“Who would I like to be in the future?”), and their relations with significant others. Using EYME, patients’ created a three-dimensional graphical “map of meanings” that they could engage with directly. VR-based EYME made abstract concepts, such as “identity” and “personal meanings”, more concrete and manageable. During therapy, the patient demonstrated significant improvement, even though six months later a follow-up check indicated a recurrence.

### 4.3. Modulating Time Experience in VR

The following number of studies are not specifically focused on the future or the past, but on how the experience of the present can be modulated through VR. Many of the studies do not involve patients with depression, but are part of the VIRTUALTIMES project [49,50,56,57,58] which aims to develop VR environments that use time manipulation to support treatments for psychological disorders such as depression. Moreover, they justify investigating time modulation in VR by its potential application to the treatment of depression.

Igarzábal et al. [56] investigated how people experience time in waiting situations. They hypothesized that boredom, thinking about time, and a slower perceived passage of time will take place in both offline and the VR waiting rooms. Since VR is typically associated with entertainment and flow states, the authors expected the time in VR to be experienced as running faster. The study involved 83 healthy students. After entering a VR waiting room, participants were told the experimenter needed to configure the system elsewhere and were asked to remain seated with the headset on. They waited for 7.5 min. Contrary to the authors’ expectations, the participants in the VR condition reported greater boredom, more time-related thoughts, and a slower passage of time than in the real waiting room settings.

Unruh et al. [50] also investigated how people experience time, while waiting in VR. They examined the relationship between virtual embodiment and time perception. The study compared offline and VR conditions, with the latter divided into avatar and no-avatar conditions. The VR waiting room was a digital reproduction of the real room used in control condition. It involved 105 participants. The experimental design was analogous to that used in the Igarzábal et al. [56]. This time, the offline condition did not differ significantly from the VR conditions, in either duration estimation, or the feeling of time passage. As predicted, participants in the avatar condition experienced a faster passage of time, and were less focused on the passing of time as compared to the offline waiting scenario.

Another Unruh et al. [70] study examined how out-of-body experiences in VR affected time perception. 44 participants were examined in two virtual disembodiment conditions (looking at their own avatar from behind or from the front). Time duration estimates were significantly shorter in the disembodied condition, than in the embodied condition, where the participants had a first-person perspective.

Landeck et al. [49] explored how the density and motion speed of a virtual tunnel generated the illusion of self-motion (vection) and modulated time perception in both the desktop and in the VR settings. The study involved 132 participants in the desktop condition and 42 in the VR condition. Participants watched a moving virtual tunnel either on a computer screen or in VR. The tunnel varied along three parameters: speed (fast vs. slow), duration (20, 30, or 40 s), and density of tunnel sections (low vs. high). Accelerated passage of time was experienced in both VR and screen conditions. Overall, participants underestimated the actual elapsed time, with greater underestimation in VR. The illusion of self-motion correlated positively with the perceived passage of time and was stronger under high-density and fast-speed conditions.

Another Landeck et al. [57] study explored how virtual objects displaying different types of motion affected time perception. They used seven types of virtual zeitgebers [54]. Landeck et al. [57] employed zeitgebers that exhibited rotary motion (e.g., a clock), irregular motion (e.g., a Newton pendulum), and linear motion (e.g., a tunnel). In a similar study, Landeck et al. [58] developed earlier work to compare a clock and an orbital pendulum. The first study [57] involved 60 participants and the second study 32 participants [58]. In both experiments, participants were exposed to all zeitgebers for 30 s. In the 2024 [58] study each object was additionally presented under three speed conditions (slow, normal and fast). Both papers indicated that zeitgebers with more visible changes (e.g., irregular motion of the pendulums) led to an accelerated feeling of time. This effect was stronger in fast conditions, as indicated by the results of the 2024 study. However, significant differences were found only between the slow vs. the normal condition, and the slow vs. the fast condition [58]. Participants underestimated the duration of time when observing zeitgebers that had irregular and dynamic motions.

Then, Ke et al. [65] explored the relationship between surrounding avatars and time perception in a VR gym. The experiment incorporated three independent variables: the motion speed of surrounding avatars performing squats (slow, medium, and fast), the intensity of the surrounding avatars’ exercises (low: only the barbell; high: barbell with weights), and the participants’ bodily movements (sitting or cycling on a stationary bike). The study involved 24 participants. Subjects underestimated time duration in all experimental conditions. The subjective feeling of time passage differed significantly depending on the exercise intensity and motion speed of surrounding avatars. The faster the motion or exercise intensity, the faster the experience of the passage of time. The authors suggested that their findings could be applied to alleviate depressive symptoms associated with the sensation of being “stuck in time” or a slowed subjective passage of time.

Moreover, Rutrecht et al. [69] used the game *Thumper* to investigate time perception in a flow state. The study involved 100 participants divided into experimental (VR condition) and a control group (desktop condition). The *Thumper* is a rhythm-based video- and VR-game, with clear goals and increasing challenges. The game successfully induced a flow state, with higher flow in the VR group. A higher-level of flow was correlated with an accelerated passage of time and reduced time awareness. However, the study did not demonstrate significant underestimation of time duration dependent on the flow state. The authors suggested that games capable of inducing a flow state could help alleviate depressive symptoms related to the subjective slowing of time experience. The *Thumper* also shares several characteristics with the game *Boson X* [72], which has been connected to a reduction in ruminative thinking.

Finally, Wang et al. [71] examined how evocative VR content modulated time perception. Participants were exposed to 1 min VR scenes featuring either evocative or conventional content. The evocative VR included three animations: lightning and thunder, a Christmas tree with flickering lights, and a moving cat. After each exposure, participants estimated the perceived duration of time. The authors highlighted the relevance of their study for interventions addressing depression or anxiety, as the evocative content modulated time perception to promote the feeling of comfort.

## 5. Results

Our scoping review indicates that the theme of temporality within VR-based interventions is wide but remains underexplored for depression. Among all studies selected for the review, only two papers explicitly addressed how VR interventions change attitudes to time in depressed patients. Colombo et al. [61] designed a VR-supported BA scheme to mitigate negative expectations, an intervention that facilitated imagination and visualization of pleasurable future activities, supported planning, and enhanced the capacity for savouring. Fernández-Alvarez et al. [62] also addressed depressed patients directly, and demonstrated that a VR-supported intervention facilitated the retrieval of their vivid and emotionally positive autobiographical memories, thereby reducing symptoms like rumination, and enabling their positive experiences in the present.

The remaining studies addressed either the experience of time, or the potential therapeutic use of VR, briefly or implicitly. Nine studies summarized in Section 4.3 focused on investigating the experience of time in VR among healthy subjects, invoking its potential therapeutic use for depression as a rationale for designing a research schema focused on time acceleration or deceleration [49,50,56,57,58,65,69,70,71]. Although VR has been shown to be able to manipulate time perception in healthy individuals, it remains an open question whether this mechanism may also be applicable to the ‘time delay’ in depression. And while these works suggest that altered time perception produced in VR may offer benefits for patients with depression, they do not provide any guidance on how to design specific interventions. Six further papers only implicitly mentioned time experience in VR therapy for patients with some form of depressive disorders [60,63,64,66,67,68]. There is a need for further research that can implement the theoretical assumptions made in these studies and their empirical findings into an actual therapeutic practice.

In order to organize the collected material, we applied the framework proposed by Montesano and Seinfield [73], which classifies therapeutic interventions in VR along three dimensions: the strategy of the intervention (exposure, training, or exploration), the focus of the therapeutic process (symptoms, attitudes, or identity exploration and flourishing), and the patient’s perspective in VR (first-, second-, third-person, or multi-perspective). Seven of the analyzed studies, applied an exposure-based strategy [49,50,56,57,58,65,71], while four adopted a skill training [60,66,67,68]. In addition, one study implemented a mixed exposure and training design [69]. Two further studies allowed participants to freely explore a virtual environment [62,63]. The studies employing exposure strategy were directed at healthy subjects, they did not explicitly aim to modify symptoms, attitudes, or self-identity, except for the one of Wang et al. [71]. Among the remaining studies, five sought to alleviate depressive symptoms [60,62,64,66,67], and one pursued both symptom reduction and attitude change [67]. The rest focused on identity exploration and personal flourishing [63,68]. Nearly all interventions, except for two, utilized a first-person perspective. García-Gutiérrez [63] and Unruh et al. [70] applied mixed-perspective designs. The former combined first- and second-person views, whereas the latter integrated first- and third-person perspectives.

The studies highlighted the psychological mechanisms that make VR an effective therapeutic tool and the practical reasons that support its integration with standard therapy. Among the reviewed studies, six papers emphasized the potential of VR to generate a state of immersion by facilitating positive engagement in the present [60,61,62,63,64,67]. This contributes to reframing the individual’s cognitive and emotional relation with the past or future events. Through immersive multimodal experiences that re-anchor attention in the present, VR offers a possibility to disrupt entrenched patterns of relating to time.

Supporting mental imagery through the use of VR is another recurring theme across studies. VR was seen as a tool that increased vividness of imagination, therefore enhancing patients’ positive attitudes towards past, present, or future [61,62,63,71]. The potential of VR to enhance one’s imagination is supported by its capacity to afford interaction with abstract entities such as time, social relations, or personal identity [63]. Effects of VR on mental imagery were observed in interventions provided for individuals with depression.

Four of the seventeen analyzed studies emphasized that VR-based interventions yielded practical benefits. VR is a relatively low-cost technology that enhances the accessibility of health care, particularly in regions where access to mental health professionals is limited [60,61,66,67]. Several studies indicated that VR systems can be flexibly adapted and personalized to meet individual patients’ needs, and could be implemented in home settings [61,62,63,66]. This could reduce the risk of stigma associated with receiving therapy at medical facilities, as well as reducing the stress associated with face-to-face interactions [50,62,66,67]. In all seventeen studies, VR interventions have been considered as complementary to other methods (e.g., standard psychotherapy, BA, tablet applications) rather than a stand-alone psychological intervention.

The analysis of the methodological aspects of the collected research reveals moderate to low reliability of their findings. We assessed the studies according to the following criteria: (a) whether the results supported the original hypotheses; (b) whether the study employed a control group; (c) whether statistically significant differences were observed between experimental and control groups; (d) whether the outcomes sustained over time; (e) whether the study was exploratory, descriptive or explanatory. Among the collected studies, three reported outcomes that contradicted the original hypotheses [56,60,68]. The majority of the remaining studies did not use a control group in their study design [58,61,62,63,65,66,67,68,70,71]. Sixteen papers indicated only short-term effects except for the study by Miller et al. [67] that indicated effects that sustained over time. All of the studies had exploratory aims, with conclusions highlighting the need for future confirmation (as shown in Table 1).

In conclusion, we found that there is a significant variation among methods and therapeutic approaches to VR interventions, with no coherent framework for studying and designing VR-based interventions that address lived temporality or time experience in depression. Most of the studies have examined how VR-based interventions can help to influence the perception of time among healthy subjects, rather than directly addressing temporality as a therapeutic focus. Many studies have highlighted the potential of using VR in therapeutic interventions to modify the perception of time, as well as its practical advantages, but all of the analyzed studies had an exploratory character and their results require better empirical grounding. While the strength and scope of conclusions that can be drawn from our study are constrained by the relatively small sample size (17 papers in total), the results at minimum demonstrate that the topic is understudied. We therefore indicate directions for future research in the next section.

## 6. Discussion and Future Directions

In view of the limitations observed, it is worth considering the possibilities for further development of research on VR-based interventions focusing on the experience of time in depression. For instance, Cavaletti [74] analyzed the use of VR in chemotherapy. Her conclusions are consistent with the studies by Landeck et al. [49,58] and Unruh et al. [50] that VR can produce a duration compression effect. This can be applied in working with depression, by creating a dynamic temporal experience. Equally important may be the choice of an interactive VR experience that actively engages the individual. However, this needs to be empirically tested, as findings from the studies conducted with healthy individuals do not necessarily translate to patient populations.

A unique feature of VR is that it enables users to actively engage with its content, rather than merely passively observing. Motor interaction, such as manipulating objects, moving through virtual spaces or performing physical exercises, enhances the sense of agency, which is significantly diminished in depression. VR-induced physical activity could thereby stimulate cognitive, emotional and motivational processes that help restore a sense of purpose and meaningful action. Since only a few of the analyzed studies exploited the action possibilities that VR offers, it is crucial to design interactive VR experiences that provide opportunities for action. Our hypothesis is that they could help patients inhabit the present moment and anticipate a more hopeful future. In case of depression, this is achievable, unlike in chemotherapy, where physical movement may be restricted. In this way, a person’s activity in VR can potentially alter their subjective experience of time and enhance the overall sense of agency and the capacity to change one’s environment.

Another outstanding feature of VR is its ability to evoke perspective-shifting experiences; however, only two of the reviewed studies applied this strategy [63,70]. VR technology allows the users to act from a first-person perspective, while also giving the opportunity to observe themselves from a third-person point of view. This strategy is applied, for instance in body-swapping experiments in VR [75] and in the study of Slater and colleagues [76], in which the participants had a dialogue with their own avatars and while embodied in Freud’s avatar. Although these features of VR can foster patients’ self-reflective capacities, as well as their relation to past and future, they were rarely used in the studies gathered for this scoping review.

Moreover, Cavaletti [74] highlights that VR is well suited to work with the experience of temporal flow, or dissolving its passage. In a flow state, not only the sense of self, but also one’s worries and concerns are said to temporarily disappear. As indicated in the analyzed studies, the sense of immersion and presence in VR fosters generation of flow states [68,69]. The higher the immersion and presence in VR, the stronger the flow experience. This applies not only to action games, but also applications that require a higher level of reflection, such as those focused on therapeutic interventions of time and identity.

Finally, according to Fuchs [77], the subjective experience of time in depression is characterized by standing still, whereas the “world-time” passes by. This discrepancy leads to the desynchronization between the patient’s lived temporality and the world. As described in Section 2.1, Fuchs proposed five principles for resynchronizing therapy as a strategy to address temporal disturbances in depression. The studies we analyzed show a clear alignment with some of the Fuchs’ guidelines. Moreover, the stages identified by Fuchs offer a valuable framework that can inform both the design and the implementation of future therapeutic interventions, which we will address now.

The first stage in Fuchs’ resynchronizing strategy is a recovery period, gradual readaptation to social rhythms with minimal pressure. The examined body of research shows that in the “timeout” period VR interventions can offer therapeutic benefits either through evoking a state of flow, temporarily increasing the internal pace of time [49,56,69] or through delivering immersive mindfulness exercises [66,67,68]. A possible extension of these strategies involves creating a personal safe space in a drawing app like OpenBrush, allowing individuals to define, visualize, imagine and revisit their safe space in VR. Another option is to use existing calming 360° YouTube videos or meditation apps like Maloka, or Flowborne VR, with biofeedback.

The second set of guidelines for resynchronization emphasizes the need for regularities and rhythms in everyday life that help to tune up distorted lived temporality with the external timing. The reviewed studies indicate that VR supports patients with scheduling activities and enhances their time orientation [60,61,64]. VR can support scheduling practice by encouraging patients to engage in activities within comfortable, immersive environments, or to participate in shared activities, such as virtual group therapy offered on platforms like Innerworld.

The third phase of the resynchronization supports future orientation. The patients are encouraged to define and start working toward achievable goals. Findings in our scoping review demonstrate that VR supports simple actions (see Section 4.2), helping patients expand their sensorimotor space, and visualize or imagine a hopeful future [61,63,67]. VR environments enable not only the observation but also the interaction with desired future scenarios. Additionally, flexible perspective shifts in VR enhance the patients’ self-reflective capacities [63,70].

During the fourth step in the resynchronization process, the patients can receive personalized stimulation suited to their current state. Several reviewed studies highlight the potential of VR to create personalized interventions that calibrate the intensity of the VR experience to the patients’ needs [62,66,67]. For instance, therapy may begin with non-interactive 360° immersive videos and gradually progress toward higher levels of interaction or task engagement.

Finally, Fuchs recommends restoring disrupted temporal and social rhythms to help patients in acute depression and grief regain a sense of continuity by re-establishing meaningful social connections. Although VR experiences can clearly accompany this process, none of the studies we reviewed specifically addressed grief, and only one focused on acute depressive disorder (In Huang et al. [64] study patients received average 22 in Hamilton Depression Rating Scale). A possible VR intervention at this stage could create a psychoeducational experience by visualizing stages of grief to support the patients’ readjustment process. Another option is an active VR experience in which individuals can create personal narratives of grief, using images or photos to reflect on their transformation.

## 7. Conclusions

The aim of this scoping review was to examine how VR interventions address the temporal dimensions of patients’ experiences in depressive episodes. The study pursued three specific objectives. First, it aimed to organize the existing body of research on temporality in VR-based interventions for depression. Second, it sought to highlight key factors that modulate time experience during VR interventions in depression. Third, it aimed to enlist limitations of the existing studies and to indicate directions for future research. The added value of our approach lies in integrating the topic of the experience of time in depression with applications of modern technology.

The analysis presented in the paper reveals that despite its clinical significance, the area remains understudied. We found that, while the existing literature acknowledges time distortion as a significant feature of depression, only a few studies explicitly target temporality in VR-based therapeutic interventions. Among the 17 papers gathered for analysis, only two directly tackled the issue of time. Nevertheless, lived temporalities and time experience were often implicitly dealt with in VR interventions for depression. We identified three clusters of research according to the dimension of time that were either explicitly or implicitly addressed: interventions supporting engagement with the present (n = 4), interventions fostering orienting towards the future (n = 4), and modulating time experience in VR (n = 9). Our review indicates that the effectiveness of VR interventions stems from its capacity to generate immersive experiences and scaffold vivid imagination. Immersion and enhanced imagination supported patients in reconnecting with meaningful past events, cherishing the present moment, and anticipating the future with hope.

Our scoping review showcases that VR holds significant potential to change the perception of time in both healthy populations and depressed patients. However, currently there is no coherent body of literature demonstrating how to design interventions that target time experience in depressed patients specifically. The mechanisms discussed in exploratory research on healthy individuals (in Section 4.3) may not apply to clinical populations, and should be systemically tested in patients with depression to provide more reliable conclusions. Since the results of the studies on healthy subjects cannot be directly extended to clinical populations of patients with depression, the literature offers limited guidance for the design of specific therapeutic interventions targeting patients in depression.

We have also highlighted that VR technology enables experiences otherwise inaccessible in real life. It allows users to shift between first-, second-, and third-person perspectives. While this appears to be a promising strategy, only two studies in the reviewed sample applied such designs. Similarly, although VR affords interaction with abstract constructs, including time, social relations, or personal identity, this strategy was applied only in one study. Thus, even though VR offers significant potential to be used interactively (i.e., engaging in physical movement or object manipulation), the existing research rarely utilizes this feature. These are the underestimated and promising possibilities of VR, that should be the focus of future research.

Our analysis further suggests that the results of the studies are of moderate to low reliability. The majority of the research was exploratory, with temporary effects, and often lacked control groups, which limits their empirical validation and reproducibility. Considerable methodological diversity further hinders meaningful comparison across studies. Methods used to measure time perception varied across studies (i.e., subjective reports, time estimations, implicit suggestions in study-specific questions), which made data integration difficult. The absence of standardized therapeutic frameworks constrains consistent application of the conclusions of the analyzed studies.

Given the methodological limitations identified in the collected studies, the conclusions drawn from our scoping review must also remain tentative and preliminary. The limitations indicated may stem from insufficient collaboration between theorists and practitioners and from the novelty of VR technologies. Therefore, there is a need for more controlled studies and long-term follow-up assessments to provide more reliable clinical evidence for the use of VR interventions for temporality in depression. More research is also needed to enhance dialogue between theorists and practitioners that could establish coherent design principles, integrating diverse methodologies and theoretical perspectives. Advancing this field will require the development and validation of standardized protocols and outcome measures that directly assess temporal processes in VR.

Future research should therefore focus on developing coherent experimental designs that explicitly address time disturbances in depression. Such frameworks could integrate phenomenological insights with empirical validation and technological possibilities that VR offers. This would support the development of interventions not just to distract or relieve suffering, but to re-synchronize the depressed individual’s temporal experience with the world.

## Data Availability

The original data presented in the study are openly available in Open Science Framework at the following URL: https://osf.io/syjr7/overview?view_only=840798c72be94016bf92525ebb177d80, accessed on 3 January 2026.

## Abbreviations

The following abbreviations are used in this manuscript:

VR	Virtual Reality
MDD	Major Depressive Disorder
DSM-5-TR	Diagnostic and Statistical Manual of Mental Disorders, Fifth Edition, Text Revision
CBT	Cognitive Behavioural Therapy
PTSD	Post-traumatic stress disorder
PRISMA	Preferred Reporting Items for Systematic Reviews and Meta-Analyses
BA	Behavioural Activation
EYME	Explore Your Meanings
WMT	Working Memory Training

## References

[1] World Health Organization (2025). Depressive Disorder (Depression).

[2] Vogel D.H.V., Krämer K., Schoofs T., Kupke C., Vogeley K. (2018). Disturbed experience of time in depression—Evidence from content analysis. Front. Hum. Neurosci..

[3] Moskalewicz M., Schwartz M.A. (2020). Temporal experience in mania. Phenomenol. Cogn. Sci..

[4] Fuchs T. (2014). Psychopathology of depression and mania: Symptoms, phenomena and syndromes. J. Psychopathol..

[5] Fusar-Poli P., Estradé A., Stanghellini G., Esposito C.M., Rosfort R., Mancini M., Norman P., Cullen J., Adesina M., Jimenez G.B. (2023). The lived experience of depression: A bottom-up review co-written by experts by experience and academics. World Psychiatry.

[6] Pedram S., Piatkowski T. (2025). Exploring the potential of virtual reality (VR) in mental healthcare: A systematic literature review. Virtual Real..

[7] Spytska L. (2024). The use of virtual reality in the treatment of mental disorders such as phobias and post-traumatic stress disorder. SSM—Ment. Health.

[8] Bell I.H., Nicholas J., Alvarez-Jimenez M., Thompson A., Valmaggia L. (2020). Virtual reality as a clinical tool in mental health research and practice. Dialogues Clin. Neurosci..

[9] Ugwuoke J.C., Akande J.O. (2024). Digital Media and the Changing Mode of Intervention Delivery: Does Intervention Delivered Online Produce the Same Effect as that Delivered Face-to-Face?. Ianna J. Interdiscip. Stud..

[10] Almuqrin A., Hammoud R., Terbagou I., Tognin S., Mechelli A. (2025). Smartphone apps for mental health: Systematic review of the literature and five recommendations for clinical translation. BMJ Open.

[11] Omisore O.M., Odenigbo I., Orji J., Beltran A.I.H., Meier S., Baghaei N., Orji R. (2024). Extended Reality for Mental Health Evaluation: Scoping Review. JMIR Serious Games.

[12] Bell I.H., Pot-Kolder R., Rizzo A., Rus-Calafell M., Cardi V., Cella M., Ward T., Riches S., Reinoso M., Thompson A. (2024). Advances in the use of virtual reality to treat mental health conditions. Nat. Rev. Psychol..

[13] Jingili N., Oyelere S.S., Nyström M.B.T., Anyshchenko L. (2023). A systematic review on the efficacy of virtual reality and gamification interventions for managing anxiety and depression. Front. Digit. Health.

[14] Dilgul M., Hickling L.M., Antonie D., Priebe S., Bird V.J. (2021). Virtual Reality Group Therapy for the Treatment of Depression: A Qualitative Study on Stakeholder Perspectives. Front. Virtual Real..

[15] Ilioudi M., Wallström S., Steingrimsson S., Lindner P., Thunström A.O., Ali L. (2023). Patient experience of a virtual reality calm room in a psychiatric inpatient care setting in Sweden: A qualitative study with inpatients. BMJ Open.

[16] Freher N.K., Van Bennekom M., Bexkens A., Veling W., Bockting C.L. (2025). Virtual Reality in the treatment of depression; what therapeutic strategies does VR target?. J. Affect. Disord. Rep..

[17] Singh A.D., Pathak A., Gupta N., Vedvrat N. Neuro-Feedback VR: Real-Time Emotion Manipulation and adaptive Environments for enhanced performance and therapy. Proceedings of the 2025 First International Conference on Advances in Computer Science, Electrical, Electronics, and Communication Technologies (CE2CT).

[18] Cieślik B., Mazurek J., Rutkowski S., Kiper P., Turolla A., Szczepańska-Gieracha J. (2020). Virtual reality in psychiatric disorders: A systematic review of reviews. Complement. Ther. Med..

[19] Riva G., Wiederhold B.K., Mantovani F. (2019). Neuroscience of virtual Reality: From virtual exposure to embodied medicine. Cyberpsychol. Behav. Soc. Netw..

[20] Mishkind M.C., Norr A.M., Katz A.C., Reger G.M. (2017). Review of Virtual Reality Treatment in Psychiatry: Evidence versus current diffusion and use. Curr. Psychiatry Rep..

[21] Immanual R., Sanjana N., Sangeetha S., Kirthika K.M., Soufiane B.O., Chakraborty C., Unhelkar B. (2025). Virtual Reality Therapy for Mental Disorder. Augmented Wellness.

[22] Wiebe A., Kannen K., Selaskowski B., Mehren A., Thöne A., Pramme L., Blumenthal N., Li M., Asché L., Jonas S. (2022). Virtual reality in the diagnostic and therapy for mental disorders: A systematic review. Clin. Psychol. Rev..

[23] Chalmers D.J. (2022). Reality+: Virtual Worlds and the Problems of Philosophy.

[24] Slater M., Banakou D., Beacco A., Gallego J., Macia-Varela F., Oliva R. (2022). A Separate Reality: An Update on Place Illusion and Plausibility in Virtual Reality. Front. Virtual Real..

[25] Csikszentmihalyi M. (1990). Flow: The Psychology of Optimal Experience. J. Leis. Res..

[26] Ratcliffe M. (2015). Experiences of Depression. A Study in Phenomenology.

[27] Ghaemi S.N. (2007). Feeling and time: The phenomenology of mood disorders, depressive realism, and existential psychotherapy. Schizophr. Bull..

[28] Gallagher S. (2012). Time, emotion, and depression. Emot. Rev..

[29] Droit-Volet S. (2013). Time perception, emotions and mood disorders. J. Physiol..

[30] Wyrick R.A. (1977). Time experience during depression. Arch. Gen. Psychiatry.

[31] Thönes S., Oberfeld D. (2015). Time perception in depression: A meta-analysis. J. Affect. Disord..

[32] Liu P., Guo H., Ma R., Liu S., Wang X., Zhao K., Tan Y., Tan S., Yang F., Wang Z. (2022). Identifying the difference in time perception between major depressive disorder and bipolar depression through a temporal bisection task. PLoS ONE.

[33] Khadem H., Shahidi S., Zarani F., Panaghi L. (2022). Mood changes in bipolar disorder: An interpretive phenomenological analysis of the space-time experience. Res. Sq..

[34] Stanghellini G., Ballerini M., Presenza S., Mancini M., Georg N., Cutting J. (2017). Abnormal time experiences in major depression: An empirical qualitative study. Psychopathology.

[35] Minkowski E. (1970). Lived Time: Phenomenological and Psychopathological.

[36] Rowland D.P., Casey L.M., Ganapathy A., Cassimatis M., Clough B.A. (2021). A Decade in Review: A Systematic Review of Virtual Reality Interventions for Emotional Disorders. Psychosoc. Interv..

[37] Miloff A., Lindner P., Hamilton W., Reuterskiöld L., Andersson G., Carlbring P. (2016). Single-session gamified virtual reality exposure therapy for spider phobia. Trials.

[38] Dilgul M., Martinez J., Laxhman N., Priebe S., Bird V. (2020). Cognitive behavioural therapy in virtual reality treatments across mental health conditions: A systematic review. Consort. Psychiatr..

[39] Lindner P., Hamilton W., Miloff A., Carlbring P. (2019). How to Treat Depression with Low-Intensity Virtual Reality Interventions. Front. Psychiatry.

[40] Cai H., Wang Z., Zhang Y., Chen Y., Hu B. A virtual-reality based Neurofeedback Game Framework for depression rehabilitation using pervasive three-electrode EEG Collector. Proceedings of the 12th Chinese Conference on Computer Supported Cooperative Work and Social Computing.

[41] Kaup K.K., Vasser M., Tulver K., Munk M., Pikamäe J., Aru J. (2023). Psychedelic replications in virtual reality and their potential as a therapeutic instrument: An open-label feasibility study. Front. Psychiatry.

[42] Li J., Theng Y., Foo S. (2014). Game-Based Digital Interventions for Depression therapy: A Systematic Review and Meta-Analysis. Cyberpsychol. Behav. Soc. Netw..

[43] Freeman D., Reeve S., Robinson A., Ehlers A., Clark D., Spanlang B., Slater M. (2017). Virtual reality in the assessment, understanding, and treatment of mental health disorders. Psychol. Med..

[44] Lindner P. (2020). Better, Virtually: The Past, Present, and Future of Virtual Reality Cognitive Behavior Therapy. Int. J. Cogn. Ther..

[45] Schneider S.M., Kisby C.K., Flint E.P. (2011). Effect of virtual reality on time perception in patients receiving chemotherapy. Support. Care Cancer.

[46] Mullen G., Davidenko N. (2021). Time compression in virtual reality. Timing Time Percept..

[47] Rogers K., Milo M., Weber M., Nacke L.E. The potential disconnect between time perception and immersion: Effects of music on VR player experience. Proceedings of the Annual Symposium on Computer-Human Interaction in Play.

[48] Lugrin J.-L., Unruh F., Landeck M., Lamour yoan Latoschik M.E., Vogeley K., Wittmann M. Experiencing waiting time in virtual reality. Proceedings of the 25th ACM Symposium on Virtual Reality Software and Technology.

[49] Landeck M., Igarzábal F.A., Unruh F., Habenicht H., Khoshnoud S., Wittmann M., Lugrin J., Latoschik M.E. (2023). Journey through a virtual tunnel: Simulated motion and its effects on the experience of time. Front. Virtual Real..

[50] Unruh F., Landeck M., Oberdörfer S., Lugrin J., Latoschik M.E. (2021). The influence of avatar embodiment on time perception—Towards VR for Time-Based Therapy. Front. Virtual Real..

[51] Unruh F., Vogel D., Landeck M., Lugrin J., Latoschik M.E. (2023). Body and Time: Virtual Embodiment and its Effect on Time Perception. IEEE Trans. Vis. Comput. Graph..

[52] Mizoguchi S., Matsumoto K., Mizuho T., Narumi T. (2023). Effect of avatar anthropomorphism on bodily awareness and time estimation in virtual reality. ACM Symp. Appl. Percept..

[53] Sabat M., Haładus B., Klincewicz M., Nalepa G.J. (2022). Cognitive load, fatigue and aversive simulator symptoms but not manipulated zeitgebers affect duration perception in virtual reality. Sci. Rep..

[54] Schatzschneider C., Bruder G., Steinicke F. (2016). Who turned the clock? Effects of Manipulated Zeitgebers, Cognitive Load and Immersion on Time Estimation. IEEE Trans. Vis. Comput. Graph..

[55] Ozyurt S., Ikhwan K. (2025). The Effect of Scaled Grid Patterns on Time Perception in Virtual Landscapes. J. Digit. Landsc. Archit..

[56] Igarzábal F.A., Hruby H., Witowska J., Khoshnoud S., Wittmann M. (2021). What happens while waiting in virtual reality? A comparison between a virtual and a real waiting situation concerning boredom, self-regulation, and the experience of time. Technol. Mind Behav..

[57] Landeck M., Unruh F., Lugrin J.-L., Latoschik M.E. From clocks to pendulums: A study on the influence of external moving objects on time perception in Virtual Environments. Proceedings of the 29th ACM Symposium on Virtual Reality Software and Technology.

[58] Landeck M., Unruh F., Lugrin J., Latoschik M.E. (2024). Object Motion Manipulation and time perception in virtual reality. Front. Virtual Real..

[59] Babbie E.R. (2014). The Practice of Social Research.

[60] Buele J., Avilés-Castillo F., Del-Valle-Soto C., Varela-Aldás J., Palacios-Navarro G. (2024). Effects of a dual intervention (motor and virtual reality-based cognitive) on cognition in patients with mild cognitive impairment: A single-blind, randomized controlled trial. J. Neuroeng. Rehabil..

[61] Colombo D., Suso-Ribera C., Ortigosa-Beltrán I., Fernández-Álvarez J., García-Palacios A., Botella C. (2022). Behavioral Activation through Virtual Reality for Depression: A Single Case Experimental Design with Multiple Baselines. J. Clin. Med..

[62] Fernandez-Alvarez J., Colombo D., Suso-Ribera C., Chirico A., Serino S., Di Lernia D., Palacios A.G., Riva G., Botella C. (2021). Using virtual reality to target positive autobiographical memory in individuals with moderate-to-moderately severe depressive symptoms: A single case experimental design. Internet Interv..

[63] Garcia-Gutierrez A., Montesano A., Feixas G. (2025). Using virtual reality to promote Self-Identity reconstruction as the main focus of therapy. J. Clin. Psychol..

[64] Huang D., Yan S., Shen S., Lv S., Lai S., Zhong S., Jia Y. (2022). Effects of virtual reality working memory training on event-based prospective memory in patients with major depressive disorder. J. Psychiatr. Res..

[65] Ke B., Wang T., Yuizono T., Kanai H. (2024). Workout at a virtual gym: Surrounding avatar’s motion speed and exercise intensity effect on the user’s time perception. PLoS ONE.

[66] Mao W., Chen W., Wang Y. (2024). Effect of virtual reality-based mindfulness training model on anxiety, depression, and cancer-related fatigue in ovarian cancer patients during chemotherapy. Technol. Health Care.

[67] Miller I., Peake E., Strauss G., Vierra E., Koepsell X., Shalchi B., Padmanabhan A., Lake J. (2023). Self-Guided Digital Intervention for Depression in Adolescents: Feasibility and Preliminary Efficacy study. JMIR Form. Res..

[68] Olasz O., Erdős S., Horváth K. (2024). The effects of Virtual Reality-Based mindfulness exercises on the perception of time, psychological and physiological states of young people: A randomized crossover trial. Mindfulness.

[69] Rutrecht H., Wittmann M., Khoshnoud S., Igarzábal F.A. (2021). Time Speeds Up During Flow States: A Study in Virtual Reality with the Video Game Thumper. Timing Time Percept..

[70] Unruh F., Lugrin J., Latoschik M.E. Out-of-virtual-body experiences: Virtual disembodiment effects on time perception in VR. Proceedings of the 30th ACM Symposium on Virtual Reality Software and Technology.

[71] Wang D., Rhee C., Park J. (2025). Exploring the role of time distortion in psychological well-being: The impact of evocative VR content. Behav. Inf. Technol..

[72] Kühn S., Berna F., Lüdtke T., Gallinat J., Moritz S. (2018). Fighting Depression: Action video game play may reduce rumination and increase subjective and objective cognition in depressed patients. Front. Psychol..

[73] Montesano A., Seinfeld S. (2025). Virtual Reality in Psychotherapy: A Three-Dimensional Framework to navigate Immersive Clinical applications. J. Clin. Psychol..

[74] Cavaletti F. (2021). Virtual reality as a time-dissolving machine in distressing medical treatment. Current perspectives and future directions. Reti Saperi Linguaggi.

[75] Petkova V.I., Ehrsson H.H. (2008). If I Were You: Perceptual illusion of body swapping. PLoS ONE.

[76] Slater M., Neyret S., Johnston T., Iruretagoyena G., De La Campa Crespo M.Á., Alabèrnia-Segura M., Spanlang B., Feixas G. (2019). An experimental study of a virtual reality counselling paradigm using embodied self-dialogue. Sci. Rep..

[77] Fuchs T. (2025). Corporealized and disembodied minds: A phenomenological view of the body in melancholia and schizophrenia. Philos. Psychiatry Psychol..

